# Heparanase as a potential player in SARS-CoV-2 infection and induced coagulopathy

**DOI:** 10.1042/BSR20210290

**Published:** 2021-07-02

**Authors:** Safa Kinaneh, Iyad Khamaysi, Tony Karram, Shadi Hamoud

**Affiliations:** 1 Department of Physiology, Rappaport Faculty of Medicine, Technion, Haifa, Israel; 2 Department of Gastroenterology, Rambam Health Care Campus and Rappaport Faculty of Medicine, Technion, Haifa, Israel; 3 Department of Vascular Surgery and Kidney Transplantation, Rambam Health Care Campus and Rappaport Faculty of Medicine, Technion, Haifa, Israel; 4 Department of Internal Medicine E, Rambam Health Care Campus and Rappaport Faculty of Medicine, Technion, Haifa, Israel

**Keywords:** Coagulopathy, COVID-19, Heparanase, SARS-COV-2

## Abstract

During the current formidable COVID-19 pandemic, it is appealing to address ideas that may invoke therapeutic interventions. Clotting disorders are well recognized in patients infected with severe acute respiratory syndrome (SARS) caused by a novel coronavirus (SARS-CoV-2), which lead to severe complications that worsen the prognosis in these subjects.

Increasing evidence implicate Heparan sulfate proteoglycans (HSPGs) and Heparanase in various diseases and pathologies, including hypercoagulability states. Moreover, HSPGs and Heparanase are involved in several viral infections, in which they enhance cell entry and release of the viruses.

Herein we discuss the molecular involvement of HSPGs and heparanase in SARS-CoV-2 infection, namely cell entry and release, and the accompanied coagulopathy complications, which assumedly could be blocked by heparanase inhibitors such as Heparin and Pixatimod.

## Background

In December 2019, a severe acute respiratory syndrome (SARS) caused by a novel coronavirus (SARS-CoV-2), was described in consecutive cases in Wuhan, China, and later defined by the World Health Organization (WHO) as coronavirus disease 2019 (COVID-19) pandemic, following a rapid worldwide spread. It is well established that SARS-CoV-2 causes multiple serious complications, where the most prominent are severe acute respiratory distress syndrome (ARDS) as well as multiple organ dysfunction including heart and kidney failure and coagulopathy [1–4]. While the deleterious impact of SARS-CoV-2 on pulmonary, cardiac and renal systems has been studied extensively, the adverse effects of this virus on coagulation process is still underestimated.

## COVID-19 and coagulation

Patients with COVID-19 exhibit clotting disorders that adversely affect the prognosis of the disease, and result in higher mortality rates [5–7]. Numerous studies have shown that abnormal coagulation markers, particularly markedly elevated d-dimer, fibrin degradation product (FDP), prolonged prothrombin time, and thrombocytopenia are common in severe patients or non-survivors of COVID-19 [8,9]. Indeed, patients infected by this novel coronavirus are at higher risk for overt disseminated intravascular coagulation (DIC) [1,8,10]. The pathogenesis of hypercoagulability in COVID-19 is not completely understood. However, excessive systemic inflammatory process, platelet activation, blood stasis in immobilized patients, and endothelial dysfunction are among possible etiologic factors that may induce coagulation abnormalities in COVID-19 patients [11–15]. Recent studies (some are observational) had documented lower mortality rate in COVID-19 patients who received anticoagulants in different regimens and doses—both prophylactic and treatment [16].

Similar dysregulations of coagulation system manifested in other coronavirus infections, such as Severe Acute Respiratory Syndrome Coronavirus (SARS-CoV-1) and Middle East Respiratory Syndrome Coronavirus (MERS-CoV) [17], suggesting a common downstream pathway underlying COVID-19-induced serious coagulation complication. Unfortunately, the mechanisms responsible for this phenomenon are poorly characterized. One of the potential systems that may play a crucial role in the exaggerated coagulation characterizing COVID-19 is the Heparan sulfate proteoglycan (HSPG) and Heparanase system. In this commentary, we will refer to potential evidences about the involvement of HSPGs and heparanase in COVID-19-induced coagulopathy, infectivity of SARS-CoV-2 and viral cell release.

## HSPGs and heparanase

HSPGs are ubiquitous constituents of the cell surface and the extracellular matrix (ECM). These macromolecules are largely responsible for binding various proteins, hormones, cytokines, and growth factors to their binding sites on the cell surface, where they exert cardinal functions related to cell–ECM interactions [18–20]. Heparanase, an endo-β-d-glucuronidase, is the only enzyme in mammals that degrades heparan sulfate (HS) chains of HSPGs [21–24]. Heparanase is involved in a wide variety of pathological processes and diseases, where elevated levels of heparanase were demonstrated, including inflammatory and infectious processes [25,26]. In addition, higher heparanase levels were measured in several malignancies [27–30], where the higher abundance of heparanase was associated with more aggressive and advanced disease, besides the occurrence of more disease-related complications [31].

## Heparanase and coagulation

Tissue factor (TF), a transmembrane protein, is the main cellular initiator of blood coagulation, where it is expressed in most body cells except blood and endothelial cells. However, in certain conditions associated with high levels of angiotensin II, TF expression is evident also in endothelial cells [33]. TF functions as a receptor and cofactor of plasma factor VII, where together they activate factor X and subsequently the coagulation cascade upon disturbance of vascular integrity [34]. TF pathway inhibitor (TFPI), a multivalent Kunitz-type plasma proteinase inhibitor, is the only endogenous modulator of TF, and is localized to cell surface of endothelial and tumor cells. Several studies showed increased plasma TFPI concentrations in myocardial infarction patients [35,36] and disseminated intravascular coagulation [37].

Degradation of HS by heparanase results in ECM remodeling and release of numerous sequestered components involved in several physiological and pathophysiological processes including blood coagulation [38]. For instance, overexpressing heparanase by transfected cells or adding exogenous heparanase was accompanied by increased levels of TFPI in cell medium [39]. Likewise, heparanase transgenic mice have higher TFPI concentration in their plasma as compared with wildtype (WT) mice [39]. Another set of experiments demonstrated that TFPI release from cell surface following heparanase addition correlated with enhanced TF activity [19]. Moreover, the same research group demonstrated that heparanase directly interacts with TF, thus facilitating factor Xa formation in the presence of TF/VIIa complex. Interestingly, heparin administration provoked disruption of TF–heparanase interaction and eventually abolished the pro-coagulant effects of heparanase, indicating another aspect of heparin anticoagulant activity, apart from enhancing anti-thrombin activity [40]. Based on former reports, the effect of heparin on heparanase is expected to be also applicable for other compounds, including low-molecular weight heparins (LMWHs) and other derivatives [41–43].

Considering the platelets, the essential component in thrombus formation, thrombin formed following activation of the coagulation system was shown to be further inducing heparanase release from platelets [44]. Cui et al. demonstrated that platelets of heparanase transgenic mice had a stronger adhesion activity as compared with WT mice [45], emphasizing heparanase pro-coagulant effect.

**Figure 1:**
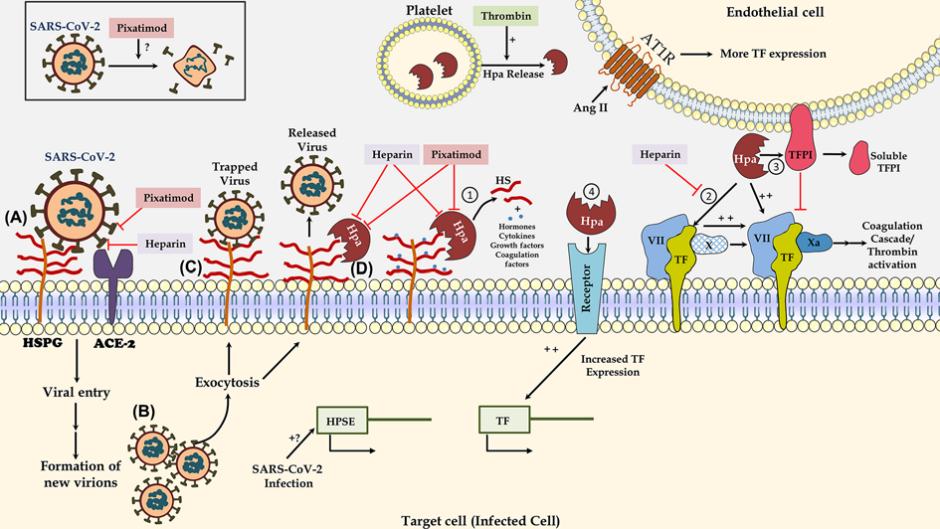
Involvement of heparanase and HSPG in SARS-CoV-2 infection and coagulation (**A**) SARS-CoV-2 binds to HSPGs thus facilitating its interaction with its homing receptor, namely ACE2, on cell surface of the infected cell. (**B**) Subsequently, the virus is internalized into the cell where it utilizes the host cells’ machinery in order to amplify itself and eventually newly replicated viruses are released. (**C**) Released viruses are trapped by HSPGs which hinder their ability to spread to neighboring cells. However, (**D**) enhanced Heparanase (Hpa) enzymatic activity induces HS degradation thus resulting into viral release and spread. Abbreviation: ACE2, angiotensin-converting enzyme 2. Heparanase acts as pro-coagulant in several ways: (**1**) Following heparanase enzymatic activity, HS is cleaved and the anchored components including coagulation factors are released. (**2**) Heparanase directly interact with TF, thus facilitating factor Xa formation in the presence of TF/VIIa complex. (**3**) Heparanase interaction with TFPI, a TF modulator, on endothelial cells results in TFPI release from cell surface, thus eliminating its TF inhibition, and finally, (**4**) Heparanase induces TF expression. Thrombin induces platelet heparanase release and so augmenting coagulation. Circulating Angiotensin II by binding to AT1 receptor leads to up-regulation of TF expression on endothelial cells. HS memetic molecules, such as Heparin and Pixatimod, inhibit heparanase along pro-coagulation activity. Moreover, these inhibitors interact with RBD on S1 of SARS-CoV-2 thus block viral cell entry. Virucidal activity of Pixatimod, verified in several viral infections, is still questionable in SARS-CoV-2 infection. Although many viral infections results in overexpression of heparanase, this still vague in SARS-CoV-2 infections. Abbreviation: RBD, receptor-binding domain.

Collectively, independent of its enzymatic activity, heparanase was shown to augment coagulation by induction of TF expression and activity [19], increasing TFPI dissociation from cell surface [39], and directly interacting with TF [40] (Figure 1), thus heparanase inhibition is supposed to attenuate these effects and eventually interfere with its procoagulant effects.

## Heparanase and HSPGs in viral infection (including coronaviruses)

In addition to their natural function in binding a variety of extracellular ligands, HSPGs serve as a binding receptor for multiple human viruses, including coronaviruses [46–51], making it a target of many studies aiming at blocking this initial interaction, thus impede viral entry [52–55].

Although it is a member of α-coronavirus family, human coronavirus NL63 (HcoV-NL63) was shown to bind HSPG on cell surface, thus enhancing the virus infection [56]. Moreover, it has been reported that other coronaviruses, including SARS-CoV-1, employ HSPG for adhesion and cell entry [57–59]. These findings suggest that cell surface HS increases viral density at the cell surface and facilitate the interaction between CoV-NL63 or SARS-CoV, and Angiotensin-Converting Enzyme 2 (ACE2), the homing receptor for these viruses and their cellular entry [60].

Similar to HSPG, heparanase is deeply involved in viral infection and release. Most studies in this context were performed on herpes simplex virus-1 (HSV-1) infection. Subsequent to HSV-1 infection, HS levels on cell surfaces were dramatically decreased, along heparanase up-regulation [61]. Similar observations were detected in HSV-2 infection [62] and a variety of other viral strains [63–65].

A recent study showed that knockdown of heparanase by using shRNA in mouse and human corneal epithelial cells resulted in significant decrease in HSV-1 release. Seemingly, overexpression of heparanase in these systems resulted in profound viral release [61]. Influenza virus uses analogous mechanisms, where it encodes its own neuraminidase enzyme that facilitates viral detachment and spread after cleaving cell surface sialic acid residues [66]. This observation is contrary to HSV-1 that has no similar enzyme capable of degrading HS, but instead drives the host cell to express more heparanase.

Concerning SARS-CoV-2, a recent study by Buijsers et al. demonstrated higher circulatory heparanase levels and activity in COVID-19 patients as compared with healthy controls [32]. Moreover, these authors found that the disease severity was positively correlated with serum levels of heparanase, HS and IL-6 [32]. Another study by Clausen et al. provided evidence that HSPGs enhance the binding of SARS-CoV-2 to ACE2 through the receptor-binding domain (RBD), and heparin and other anticoagulant agents blocked this interaction [67]. However, additional studies are needed in this context.

Collectively, HSPGs and heparanase are shown to be implicated in the pathogenesis of several unrelated viruses including coronaviruses infections. Given that heparanase overexpression was detected in many unrelated viral systems and recently in COVID-19 [32], this may indicate that up-regulation of heparanase is a common strategy among viral species to increase spread and transmission, and the most important point is that this strategy is already confirmed to be adopted by SARS-CoV-2.

## Heparanase inhibition as a COVID-19 therapeutic maneuver

After saying that, heparanase inhibition using HS mimicking compounds, anticoagulant heparin, or any other inhibitors, may abolish the deleterious effects of heparanase and HSPGs both on coagulation system, by interfering with TF–heparanase interaction, and blocking viral–cell attachment and cell-to-cell spread.

In this context, heparin, a highly sulfated form of HS that inhibits HS degradation by heparanase, blocked cell binding and invasion by SARS-CoV-2, as it bound to spike (S) 1 RBD leading to conformational change [68]. Noteworthy, sequence analysis of the glycosylated S protein of SARS-CoV-2 revealed that RBD of S1 contained HS-binding sites [69–71]. Similarly, heparin was previously shown to partially block infectivity of SARS-associated coronavirus strain HSR1 [72].

At the clinical level, administration of heparin or LMWH (Enoxaparin) to COVID-19 patients improved their physical condition and survival rate [73]. In a study that included 449 severe COVID-19 patients in Wuhan, prophylactic dose heparin therapy significantly reduced mortality [74]. More specifically, Buijsers et al. showed that prophylactic LMWH reduced heparanase activity in COVID-19 patients [32], a finding that may explain the reduction in SARS-CoV-2 entry to cells following this treatment. Another study that supports this notion is the study by Billet et al. who demonstrated that administration of enoxaparin, unfractionated heparin, and apixaban to severe cases of COVID-19 reduced the mortality rate, most probably due to lower thromboembolic complications, while it could be acceptable to postulate a role of inhibiting heparanase activity in these patients [75].

Pixatimod (PG545), an HS memetic compound, is known as a potent heparanase inhibitor [76]. Apart from its anticancer [77,78], anti-inflammatory [25,79,80], antioxidative [81], and anti-atherosclerotic [82] activities, Pixatimod was previously shown to have antiviral activity to a number of viruses that utilize HS as binding and entry receptor [83–86]. In addition, Pixatimod was shown to have virucidal activity on HSV-1, where viral lipid envelope is disrupted by its lipophilic steroid side chain [84,87].

Interestingly, Pixatimod was recently demonstrated to interact with RBD of S1 of SARS-CoV-2 and thus inhibited viral attachment and invasion in ACE2-expressing vero-cells [88]. Since Pixatimod strongly inhibits heparanase, these results support a deleterious role of this enzyme in the pathogenesis of COVID-19 manifestations, especially coagulopathy, and suggest a therapeutic potential of Heparanase inhibitors as antiviral and anticoagulative agents during this disease state. NCT identifier search revealed no studies dealing with the effect of Pixatimod on COVID-19 patients. Additional heparanase inhibitors are under clinical and basic science research, like SST0001, but NCT identifier search revealed no studies for these compounds in COVID-19 patients. Nevertheless, as for 26 April 2021, we found 132 ongoing (129)/completed (*n*=3) studies that deal with the effects of different anticoagulants on COVID-19 subjects, and included unfractionated heparin, LMWHs, and direct oral anticoagulants (DOACs)—namely Rivaroxaban (11 studies), Apixaban (7 studies), and Edoxaban (1 study).

In conclusion, herein we summarized the current knowledge regarding the role of heparanase in the pathogenesis of several diseases and the favorable effect of its inhibition in a wide variety of pathologic processes. It is tempting to explore potential role of heparanase in these clinical settings including COVID-19, and to examine the effect of heparanase inhibition in SARS-CoV2-infected subjects, which might be beneficial to heal coagulation system and to attenuate viral attachment and spread, and subsequently to improve the prognosis of infected subjects.

## Data Availability

All data generated or analyzed during the present study are included in the review.

## Abbreviations

ACE2: angiotensin-converting enzyme 2
COVID-19: coronavirus disease 2019
ECM: extracellular matrix
HSPG: heparan sulfate proteoglycan
HSV-1: herpes simplex virus-1
LMWH: low-molecular weight heparin
RBD: receptor-binding domain
SARS-CoV: severe acute respiratory syndrome -corona virus
TF: tissue factor
TFPI: tissue factor pathway inhibitor
WT: wild-type
